# Short-term Mortality Outcomes of HIV-Associated Cryptococcal Meningitis in Antiretroviral Therapy–Naïve and –Experienced Patients in Sub-Saharan Africa

**DOI:** 10.1093/ofid/ofab397

**Published:** 2021-07-28

**Authors:** Newton Kalata, Jayne Ellis, Cecilia Kanyama, Charles Kuoanfank, Elvis Temfack, Sayoki Mfinanga, Sokoine Lesikari, Duncan Chanda, Shabir Lakhi, Tinashe Nyazika, Adrienne K Chan, Joep J van Oosterhout, Tao Chen, Mina C Hosseinipour, Olivier Lortholary, Duolao Wang, Shabbar Jaffar, Angela Loyse, Robert S Heyderman, Thomas S Harrison, Síle F Molloy

**Affiliations:** 1 Malawi Liverpool Wellcome Trust Clinical Research Programme, Blantyre, Malawi; 2 Division of Infection and Immunity, University College London, London, UK; 3 University of North Carolina Project, Kamuzu Central Hospital, Lilongwe, Malawi; 4 University of Dschang, Dschang, Cameroon; 5 Douala General Hospital, Douala, Cameroon; 6 Muhimbili Centre, National Institute for Medical Research, Dar Es Salaam, Tanzania; 7 University Teaching Hospital, Lusaka, Zambia; 8 Dignitas International, Zomba Central Hospital, Zomba, Malawi; 9 Sunnybrook Health Sciences Centre, University of Toronto, Toronto, Ontario, Canada; 10 Liverpool School of Tropical Medicine, Liverpool, UK; 11 Necker Pasteur Center for Infectious Diseases and Tropical Medicine, IHU Imagine, Assistance Publique–Hôpitaux de Paris, Paris, France; 12 Centre for Global Health, Institute of Infection and Immunity, St George University of London, London, UK; 13 MRC Centre for Medical Mycology, University of Exeter, Exeter, UK

**Keywords:** Antiretroviral therapy, Cryptococcal meningitis, HIV, Short-term mortality, Sub-Saharan Africa

## Abstract

**Background:**

An increasing proportion of patients with HIV-associated cryptococcal meningitis have received antiretroviral therapy (ART) before presentation. There is some evidence suggesting an increased 2-week mortality in those receiving ART for <14 days compared with those on ART for >14 days. However, presentation and outcomes for cryptococcal meningitis patients who have recently initiated ART, and those with virologic failure and/or nonadherence, are not well described.

**Methods:**

Six hundred seventy-eight adults with a first episode of cryptococcal meningitis recruited into a randomized, noninferiority, multicenter phase 3 trial in 4 Sub-Saharan countries were analyzed to compare clinical presentation and 2- and 10-week mortality outcomes between ART-naïve and -experienced patients and between patients receiving ART for varying durations before presentation.

**Results:**

Over half (56%; 381/678) the study participants diagnosed with a first episode of cryptococcal meningitis were ART-experienced. All-cause mortality was similar at 2 weeks (17% vs 20%; hazard ratio [HR], 0.85; 95% CI, 0.6–1.2; *P* = .35) and 10 weeks (38% vs 36%; HR, 1.03; 95% CI, 0.8–1.32; *P* = .82) for ART-experienced and ART-naïve patients. Among ART-experienced patients, using different cutoff points for ART duration, there were no significant differences in 2- and 10-week mortality based on duration of ART.

**Conclusions:**

In this study, there were no significant differences in mortality at 2 and 10 weeks between ART-naïve and -experienced patients and between ART-experienced patients according to duration on ART.

Cryptococcal meningitis (CM) continues to cause significant morbidity and mortality in people with HIV (PWH) despite the scale-up of antiretroviral therapy (ART) in Sub-Saharan Africa (SSA), and an increasing proportion of patients with HIV-associated CM are now presenting on ART [1]. Among ART-experienced patients, a proportion will have unmasking immune reconstitution inflammatory response syndrome (IRIS) (usually those with ART initiation ~< 3 months before presentation), and a proportion will have virological and immunological failure due to ART failure (usually those on ART ~>6 months) [2]. Although it is well recognized that early initiation of ART during induction treatment of CM results in higher mortality [3, 4], there are few data available on outcomes for CM patients recently started on ART before presentation, and guidance on ART management (continuation, switch, or interruption) in this group is based largely on expert opinion [5]. Overall mortality appears to be similar when comparing ART-experienced and ART-naïve CM patients [6–9]. However, differences in presentation and outcomes for patients with “unmasking” cryptococcal infection, where ART has been recently initiated, and for those with virologic failure and/or nonadherence are not well described.

A recent study of 605 PWH diagnosed with CM in Uganda [10] found no difference in 2-week mortality between the ART-experienced and ART-naïve groups, but there was increased short-term mortality in those receiving ART for <2 weeks compared with those on ART for >2 weeks. The study suggested the possibility that preexisting subclinical meningitis in some patients at ART initiation may drive an early unmasking IRIS and increased mortality. Here we have compared clinical presentation and short-term mortality outcomes for 678 ART-naïve and ART-experienced patients with a first episode of CM enrolled into the Advancing Cryptococcal Meningitis Treatment for Africa (ACTA) trial in 4 countries in SSA [9]. The impacts of ART duration before presentation on mortality outcomes were also compared.

## METHODS

### Study Setting and Population

From January 2013 to November 2016, 721 HIV-infected adults (≥18 years old) from centers in Malawi, Zambia, Tanzania, and Cameroon presenting with a first episode of CM were prospectively enrolled into a randomized, noninferiority, multicenter trial (ACTA) as previously described [9]. ART-experienced patients were excluded at the beginning of the trial (4% of enrollment), but an amendment allowed inclusion of this population after noting that a large number of patients exposed to ART were presenting with CM. A total of 678 participants were included in the final intention-to-treat analysis.

### Study Design

Patients were randomized to 1 of 3 treatment strategies: oral fluconazole plus flucytosine for 2 weeks, 1-week Amphotericin B (AmB)-based therapy, and standard 2 weeks AmB-based therapy [9]. Those in the AmB arms were further randomized to flucytosine or fluconazole in a 1:1 ratio, as the partner drug given with AmB. Patients received consolidation fluconazole therapy after the 2-week induction period: 800 mg until ART initiation or switch at 4 weeks and 400 mg to 10 weeks. The primary and secondary end points were all-cause mortality at 2 weeks and 10 weeks, respectively. ART exposure was defined as ever having taken ART. Information on ART status and duration on ART was ascertained by self-report (or, where appropriate, from guardians) and confirmed by review of medical records. First-line ARTs for adults were tenofovir based.

### Study Outcomes and Analysis

All-cause mortality at 2 and 10 weeks was compared based on ART status (experienced vs naïve) and ART duration at CM diagnosis. Baseline clinical and laboratory characteristics were compared across ART groups, with the chi-square or Fisher exact test, where appropriate, for categorical variables and Kruskal Wallis tests for continuous variables. Unadjusted and adjusted Cox proportional hazards models, Kaplan-Meier curves, and log-rank tests were used to examine the hazard of mortality between ART-experienced and ART-naïve patients and for those on ART for varying durations. A global test was conducted to test for the nonproportional hazard assumption on the Kaplan-Meier curves. Adjusted models included known prognostic markers for poor outcome: age, baseline cerebrospinal fluid (CSF) fungal count (quantitative cultures [QCCs] calculated as described elsewhere [11]), Glasgow Coma Score (GCS) scale, CSF white blood cell count, hemoglobin, antifungal treatment (flucytosine vs nonflucytosine regimens), and recruitment site.

A distinct cutoff for the duration a patient has been taking ART to help distinguish between patients experiencing “unmasking” CM and those with virologic failure/nonadherence has not been clearly defined. Results from Rhein et al. [10] suggested an increased mortality for patients receiving ART for ≤14 days compared with those on therapy for 15–182 days or >6 months. Therefore, we examined these time periods, and also conducted exploratory analyses to understand whether alternative time points with a cutoff of 1 month, 2 months, 3 months, and 6 months could be used to distinguish subgroups within the ART-experienced population and identify any differences in mortality. All analyses were performed in Stata, version 15 (StataCorp LP, College Station, TX, USA).

## RESULTS

### Outcomes by ART Status

**Figure 1.**
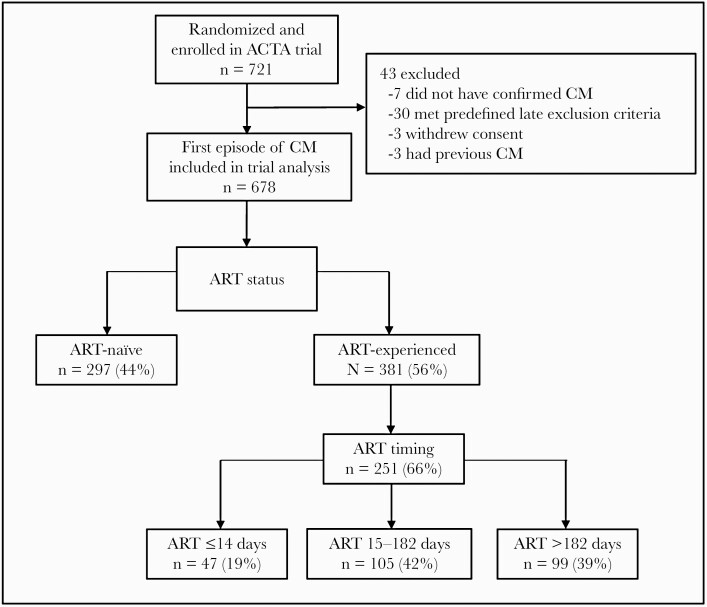
Study cohort. Abbreviations: ACTA, Advancing Cryptococcal Meningitis Treatment for Africa; ART, antiretroviral therapy; CM, cryptococcal meningitis.

A total of 678 patients were included in the study, with 56% (381) being ART-experienced at baseline (Figure 1). Overall, there was little difference in baseline demographics and clinical symptoms between ART-naïve and -experienced patients (Table 1), although both median plasma HIV viral load and median CSF fungal burden were higher for ART-naïve patients (*P* < .001). All-cause mortality was similar at 2 weeks (17% vs 20%; *P* = .39; hazard ratio [HR], 0.85; 95% CI, 0.6–1.2; *P* = .35) and 10 weeks (38% vs 36%; *P* = .64; HR, 1.03; 95% CI, 0.8–1.32; *P* = .82) for ART-experienced vs ART-naïve patients, respectively, with similar results in adjusted analyses (data not shown).

**Table 1.** Clinical Presentation and Outcomes by ART Status and ART Duration

	ART Status, No. (%)/Median (IQR)			ART Duration, No. (%)/Median (IQR)		
	ART-Naïve	ART-Experienced	*P* Value	≤14 d	>14 d	*P* Value
	(n = 297)	(n = 381)		(n = 47)	(n = 204)	
Demographics/clinical characteristics						
Male	173 (58)	217 (57)	.74	30 (64)	118 (58)	.45
Headache duration, d	14 (7–21)	14 (7–28)	.33	7 (7–20)	14 (7–30)	**.03**
Seizures (within 72 h)	54 (18)	65 (17)	.70	9 (19)	32 (16)	.56
Confusion	119 (40)	148 (39)	.75	22 (47)	74 (36)	.18
Current TB	36 (12)	62 (16)	.13	8 (17)	37 (18)	.86
Markers of HIV disease severity						
Anemia (Hb <7 g/dL)	5 (2)	13 (4)	.16	2 (4)	10 (5)	.84
CD4 count, cells/mL	25 (10–55)	28 (10–68)	.51	41 (22–83)	33 (12–78)	.10
CD4 count <100 cells/mL	250 (91)	312 (88)	.10	33 (81)	164 (86)	.38
Viral load, log_10_ copies/mL	5.4 (4.9–5.7)	4.0 (2.5–5.0)	**<.001**	3.5 (2.9–3.9)	3.8 (2.2–4.9)	.99
Markers of severe cryptococcal disease						
Age ≥50 y	34 (12)	41 (11)	.77	7 (15)	17 (8)	.17
GCS <15	76 (26)	87 (23)	.41	15 (32)	42 (21)	.10
CSF opening pressure >25 mm CSF	125 (46)	159 (45)	.73	21 (50)	94 (49)	.88
CSF WCC <5 cells/mL	156 (56)	199 (54)	.67	22 (51)	108 (55)	.64
CSF fungal count, log_10_ CFU/mL	5.2 (4.2–5.7)	4.7 (3.1–5.8)	**.001**	5.2 (3.9–5.9)	4.2 (2.7–5.4)	**.004**
Clinical management						
5FC-based antifungals	187 (63)	226 (70)	.06	35 (75)	137 (67)	.33
No. of LPs received	3 (2–3)	3 (2–3)	.9	3 (2–5)	3 (3–4)	.68
CSF clearance rate	0.34 (0.23–0.50)	0.33 (0.22–0.50)	.82	0.36 (0.27–0.63)	0.32 (0.22–0.47)	.06
All-cause mortality						
2 wk	59 (20)	66 (17)	.39	11 (23)	30 (15)	.15
10 wk	107 (36)	144 (38)	.64	16 (34)	78 (38)	.59

Abbreviations: ART, antiretroviral therapy; 5FC, flucytosine; CFU, colony-forming units; CSF, cerebrospinal fluid; GCS, Glasgow Coma Score; Hb, hemoglobin; IQR, interquartile range; LP, lumbar puncture; TB, tuberculosis; WCC, white cell count.

### Outcomes by ART Duration

**Figure 2.**
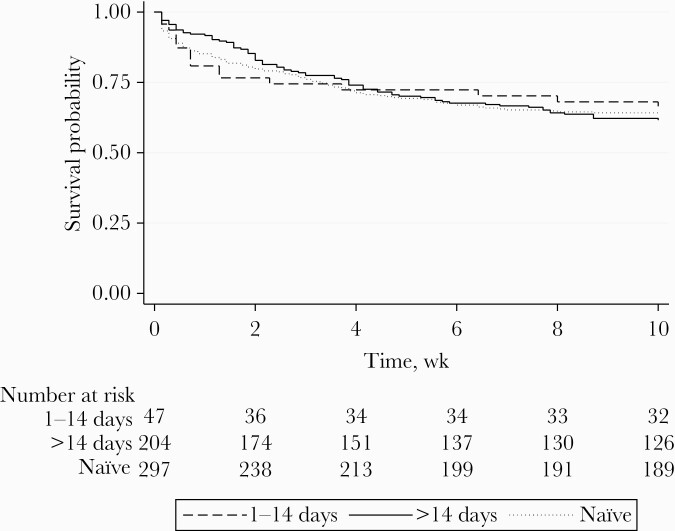
Kaplan-Meier survival plot by ART status. Abbreviation: ART, antiretroviral therapy.

Overall, 66% of patients (251/381) had data on ART duration (Figure 1). At recruitment, study participants had been on ART for a median (interquartile range) time of 12 (3–91) weeks. Nineteen percent (47/251) of patients had initiated ART within 14 days before presentation, and 61% (152/251) within 182 days (Table 1; Supplementary Table 1). There was similarity in the distribution of baseline demographics and clinical features between those taking ART for ≤14 days compared with those on ART for >14 days, though the duration of headache was shorter for those taking ART for ≤14 days (median duration, 7 days vs 14 days; *P* = .03), and CSF fungal burden was 1 log higher (Table 1). Comparing survival curves, there was weak evidence of a trend toward increased mortality in those taking ART for ≤14 days, but there was no statistically significant difference in 2-week mortality (23% vs 15%; HR, 1.70; 95% CI, 0.85–3.39; *P* = .13), and 10-week mortality was very similar (34% vs 38%; HR, 0.92; 95% CI, 0.54–1.57; *P* = .76) (Figure 2), with similar results in adjusted analyses.

Comparable results were obtained when ART duration was divided into ≤2 weeks, 15–182 days, and >182 days, and with a 1-month cutoff (Supplementary Table 1, Supplementary Figure 1). Higher viral loads and lower CD4 counts were identified for those reportedly on ART for >182 days compared with those on ART for shorter durations (Supplementary Table 1), consistent with the possibility that the former group had a higher proportion of nonadherent patients or patients who had developed ART resistance. There were no significant differences in 2- and 10-week mortality when exploring varying cutoff points for ART duration (Supplementary Table 2). Using a 1-month cutoff, 2-week mortality was 23% for those taking ART for ≤1 month vs 14% for those taking ART for >1 month (*P* = .08; HR, 1.74; 95% CI, 0.94–3.24; *P* = .07), but this trend did not hold in the adjusted analysis (adjusted HR, 1.44; 95% CI, 0.73–2.85; *P* = .30).

## DISCUSSION

Over half the patients with a first episode of CM included in this study were ART-experienced, with 14% having started ART in the 14 days before diagnosis. While ART-naïve patients had higher median HIV plasma viral loads and fungal burdens, there was no evidence for an overall difference in mortality between ART-naïve and -experienced populations at both 2 weeks and 10 weeks. In exploratory analyses according to duration on ART, there was a trend in the unadjusted analysis of increased 2-week mortality for those taking ART for <1 month compared with those taking ART for >1 month, and the survival curves suggested the possibility of an increase in short-term mortality, between 1 and 2 weeks of antifungal treatment, in those taking ART for ≤2 weeks. Overall, however, we did not identify a cutoff for ART duration that led to significant differences in short-term mortality.

Our findings differ in some respects from the report of Rhein et al. The size of our study is similar to that of Rhein and colleagues in Uganda [10], and the findings are in many respects similar in terms of the patient populations and differences, for example, in CD4 cell counts and cryptococcal colony-forming unit (CFU) counts between groups. However, we did not find a significant difference in short-term mortality in those recently started on ART before presentation with CM, or as high a 2-week mortality in those who had initiated ART within 2 weeks of presentation. This may simply be because both our studies were relatively small, with only 51 and 47 patients, respectively, within the subgroup that had initiated ART within 2 weeks of presentation.

In addition, there were some differences in practice. In the study of Rhein et al., roughly a quarter of patients who had initiated ART within 2 weeks of presentation received corticosteroids and/or had ART withheld. In the ACTA trial, conducted before publication of the Uganda data, ART was not routinely withheld among participants who had commenced ART within 6 months of presentation with meningitis and who reported and were assessed as adherent to ART. Corticosteroids were also not used in this group, in light of the results of the CryptoDex study [12], which found them not to be beneficial, including in the subgroup of patients who had initiated ART within 3 months of meningitis diagnosis.

It should be noted that results from this study were limited by missing data for a number of patients for duration of taking ART and the fact that the definition for ART exposure was broad. Therefore, the study was underpowered to detect differential mortality outcomes among the ART-experienced subgroups. This is because assessing ART adherence retrospectively is very difficult. While several measures of checking adherence are operational in most ART programs in Sub-Saharan Africa, drug adherence continues to be an important problem, with levels of noncompliance ranging between 2% and 70% [13].

Data from ongoing randomized studies may help clarify the most appropriate management for ART-experienced patients diagnosed with CM. The ongoing AMBITION-cm trial [14], and indeed data from all 3 studies combined [9, 10, 14], presents the opportunity to assess outcomes in patients on ART for various durations before meningitis. In the AMBITION-cm trial, based on a recent consensus among the investigators, including the team from Uganda, the current management strategy is to withhold ART for 4–6 weeks in those reportedly adherent and started or restarted on ART within the last 14 days, but to continue ART in those reportedly adherent and started or restarted on ART between 14 days and 6 months before presentation [5].

## Supplementary Data

Supplementary materials are available at *Open Forum Infectious Diseases* online. Consisting of data provided by the authors to benefit the reader, the posted materials are not copyedited and are the sole responsibility of the authors, so questions or comments should be addressed to the corresponding author.

ofab397_suppl_Supplementary_Materials
