# Supplementary material for: Short-term Mortality Outcomes of HIV-Associated Cryptococcal Meningitis in Antiretroviral Therapy–Naïve and –Experienced Patients in Sub-Saharan Africa
Source: Open Forum Infect Dis. 2021 Jul 28;8(10):ofab397. doi: 10.1093/ofid/ofab397 (PMC8501291; doi:10.1093/ofid/ofab397)
Supplement: ofab397_suppl_Supplementary_Materials [file ofab397_suppl_supplementary_materials.docx]

*Supplementary table 1: Clinical presentation and outcomes by ART duration at varying cut-off points*

|  | ART duration | | | | | | |
| --- | --- | --- | --- | --- | --- | --- | --- |
|  | **≤14 days**  (N=47) | **15-182 days**  (N=105) | **>182 days**  (N=99) | **p-value** | **≤1 month**  (N=75) | **>1 month**  (N=176) | **p-value** |
|  |  | **Demographics/clinical characteristics** | | |  |  |  |
| Male | 30 (64%) | 60 (57%) | 58 (59%) | 0.74 | 47 (62%) | 101 (57%) | 0.44 |
| Headache duration (days) | 7 (7 - 20) | 14 (7 - 30) | 14 (7 - 21) | 0.04 | 10 (7 - 21) | 14 (7 - 30) | 0.07 |
| Seizures (within 72 hours) | 9 (19%) | 18 (17%) | 14 (14%) | 0.72 | 32 (43%) | 64 (36%) | 0.35 |
| Confusion | 22 (47%) | 43 (41%) | 31 (31%) | 0.15 | 13 (17%) | 28 (16%) | 0.78 |
| Current TB | 8 (17%) | 22 (21%) | 15 (15%) | 0.55 | 11 (15%) | 34 (19%) | 0.38 |
| Markers of HIV disease severity and control | | | | | | | |
| Hb<7g/dl | 2 (4%) | 9 (9%) | 1 (1%) | 0.04 | 5 (7%) | 7 (4%) | 0.37 |
| CD4 count (cells/ml) | 41 (22 - 83) | 53 (20 - 85) | 21 (7 - 52) | <0.001 | 48(22 - 93) | 32(11 - 68) | 0.01 |
| CD4 count <100 cells/ml | 33 (81%) | 78 (82%) | 86 (90%) | 0.24 | 52 (79%) | 145(87%) | 0.10 |
| Viral load (log_10_ copies/ml) | 3.5 (2.9 - 3.9) | 2.6 (2.1 - 4.6) | 4.7 (2.8 - 5.3) | 0.01 | 3.2 (2.8 - 3.9) | 4.4 (2.1 - 5.0) | 0.35 |
| Markers of severe cryptococcal meningitis | | | | | | | |
| Age ≥50 (years) | 7 (15%) | 11 (11%) | 6 (6%) | 0.22 | 12 (16%) | 12 (7%) | 0.02 |
| GCS<15 | 15 (32%) | 22 (21%) | 20 (20%) | 0.25 | 21 (28%) | 36 (23%) | 0.19 |
| CSF opening pressure >25mmCSF | 21 (50%) | 44 (44%) | 50 (53%) | 0.47 | 31 (45%) | 84 (51%) | 0.43 |
| CSF WCC <5cells/ml | 22 (51%) | 55 (54%) | 53 (56%) | 0.84 | 37 (52%) | 93 (55%) | 0.65 |
| CSF fungal culture (log_10_ CFU/ml) | 5.2 (3.9 - 5.9) | 3.6 (1.8 - 5.0) | 4.9 (3.6 - 5.7) | **<0.001** | 4.8 (3 - 6) | 4.2 (3 - 5) | 0.11 |
| Clinical management | | | | | | | |
| 5FC-based antifungals | 35 (75%) | 71 (68%) | 66 (67%) | 0.62 | 54 (72%) | 118 (67%) | 0.44 |
| Number LPs received | 3 (2 - 5) | 3 (3 - 4) | 3 (3 - 5) | 0.26 | 3 (3 - 4) | 3 (3 - 4) | 0.21 |
| CSF clearance rate | 0.36  (0.26 - 0.63) | 0.32  (0.22 - 0.41) | 0.31  (0.22 - 0.47) | 0.15 | 0.34  (0.24 - 0.62) | 0.32  (0.22 - 0.47) | 0.12 |
| Mortality | | | | | | | |
| 2 weeks | 11 (23%) | 15 (14%) | 15 (15%) | 0.34 | 17 (23%) | 24 (14%) | 0.08 |
| 10 weeks | 16 (34%) | 38 (36%) | 40 (40%) | 0.71 | 30 (40%) | 64 (36%) | 0.59 |

Abbreviations: WCC: White cell count Hb: Haemoglobin CFU: Colony forming units CSF: Cerebrospinal fluid

*Supplementary Table 2: ART duration cut-off points exploratory univariable analysis*

| ART duration | 2-week mortality | p-value | 10-week mortality | p-value |
| --- | --- | --- | --- | --- |
| ≤7days  >7 days | 4 (17%)  37 (16%) | 0.89 | 6 (26%)  88 (38%) | 0.24 |
| ≤14 days  >14 days | 11 (23%)  30 (15%) | 0.15 | 16 (34%)  78 (38%) | 0.59 |
| ≤1month  >1 month | 17 (23%)  24 (14%) | 0.08 | 30 (40%)  64 (36%) | 0.59 |
| ≤2months  >2 months | 21 (21%)  20 (13%) | 0.12 | 40 (40%)  54 (36%) | 0.56 |
| ≤3 months  >3 months | 24(19%)  17(14%) | 0.35 | 47(36%)  47(39%) | 0.66 |
| ≤6months  >6 months | 26 (17%)  15 (15%) | 0.68 | 54 (36%)  40 (40%) | 0.44 |

Supplementary Figure 1: *Kaplan Meier survival plot by ART timing*
